# Stress-Preventive Management Competencies, Psychosocial Work Environments, and Affective Well-Being: A Multilevel, Multisource Investigation

**DOI:** 10.3390/ijerph15030397

**Published:** 2018-02-26

**Authors:** Stefano Toderi, Cristian Balducci

**Affiliations:** Department of Psychology, University of Bologna, Viale Berti Pichat 5, 40126 Bologna, Italy; cristian.balducci3@unibo.it

**Keywords:** work stress, supervisors’ management competencies, psychosocial working environment, affective well-being, multilevel structural equation modelling

## Abstract

The Management Competencies for Preventing and Reducing Stress at Work framework represents one of the few tailored models of leadership for work stress prevention purposes, but it has never been empirically evaluated. The aim of this study was to investigate whether supervisors’ stress-preventive management competencies, as measured by the Stress Management Competencies Indicator Tool (SMCIT), are related to employees’ affective well-being through psychosocial work environmental factors. To this end, multilevel structural equation modelling (MSEM) was developed and tested, including data provided by both supervisors and employees. Supervisors (*n* = 84) self-assessed their stress-preventive management competencies (i.e., being respectful and responsible, managing and communicating existing and future work, reasoning and managing difficult situations, and managing the individual within the team) with a previously validated reduced version of the SMCIT. The supervised employees (*n* = 584) rated job content (e.g., job demands) and work context (e.g., role clarity) psychosocial factors and their job-related affective well-being. Supervisors’ job-related affective well-being was also included in the tested model. The results revealed that the stress-preventive competencies factor was related to employees’ affective well-being through the psychosocial work environment only when the latter was operationalized by means of contextual work factors. Supervisors’ affective well-being was related to their stress-preventive competencies, but it was not related to employees’ affective well-being. We discuss the implications of the results obtained.

## 1. Introduction

The important role that leaders play in determining employees’ outcomes has been expanded from “traditional” performance-related consequences to occupational health outcomes, such as psychological well-being, work stress, cardiovascular disease, organizational safety climate, workplace accidents and injuries, and health-related behaviours [1]. In this context, a growing body of studies has applied leadership models to the domain of work stress and well-being. Skakon et al. [2] systematically reviewed the existing literature on this subject, pointing out that “… leader stress, leader behaviours and leadership style impact on employee stress and affective well-being” (p. 133). At the same time, Kelloway and Barling [1] focused on the empirical evidence concerning leadership development as an intervention in occupational health psychology, and concluded: “the available data suggest that leadership development provides occupational health psychologists with a pragmatic and effective tool” (p. 274).

Despite this strong evidence on the role of leaders in work stress prevention and the importance of action directed at leaders, research and practice on these matters have mainly transferred traditional constructs and measures of leadership to the occupational health domain. More specific and tailored theoretical frameworks and measures could provide more useful guidance for research and practice, facilitating the comparison of findings across studies. The Management Competencies for Preventing and Reducing Stress at Work framework [3,4] represents an exception in this area, since it proposes a tailored model of leadership for work stress prevention purposes and a specific measure, the Stress Management Competency Indicator Tool (SMCIT). Recently, Eurofound and the European Agency for Safety and Health at Work (EU-OSHA) [5] proposed the framework as a good practice for the development of supervisors’ behaviour in a work stress prevention context. However, to date it has never been empirically evaluated, limiting its application for practical purposes. The general aim of this study is to provide empirical support for the effectiveness of the model, allowing its use in practice for preventing stress at work.

In the next sections, we first briefly review the available knowledge on the role of supervisors and leadership as far as work stress is concerned, and the importance of supervisor development as an organizational intervention. Then, we describe in more detail the Management Competencies for Preventing and Reducing Stress at Work framework. Thirdly, based on the existing literature, we set out the hypotheses of the research, which sought to evaluate the utility of the line manager competency framework and questionnaire (SMCIT) for work-related stress.

### 1.1. The Supervisors’ Role in Employees’ Work-Related Stress and Well-Being

Today, it is widely acknowledged that leaders can influence employees’ health [1]. This influence seems to come about in different ways and with different processes, through direct and indirect paths [6]. Skakon et al. [2] highlighted three kinds of direct effects exerted by supervisors on employees’ affective well-being and stress: (1) supervisors can transmit their level of stress to employees simply by interacting with them and through a crossover contagion process [7]; (2) supervisors can directly influence employees’ well-being through their behaviours (e.g., support, consideration, acting with integrity, etc.) and the quality of the relationship; and (3) supervisors can affect employees’ well-being and stress through the leadership style adopted. Skakon et al.’s literature review strongly supported the existence of the first two effects. Regarding the role of leadership style, the results obtained were mixed, showing a positive effect of transformational leadership, but an inconsistent effect of the transactional type. The authors concluded by recommending that future studies examine the processes linking leaders with employee stress and well-being. In fact, supervisors can influence employees’ stress and well-being also indirectly through their influence on the work environment and impact on the presence/absence of psychosocial hazards [6]. 

Research on the mediating role of the work environment has clearly shown that perceptions of having a meaningful job [8,9], role clarity, and opportunities for development [9], mediate the relationship between transformational leadership and employees’ wellbeing. More recently, Lornudd et al. [10] noted that previous research mainly focused on transformational leadership and little is known about the role of alternative leadership models. They consequently focused on production-, employee- and change-oriented leadership [11] and studied the mediating role of demand and control in determining five distress outcomes (i.e., disengagement, exhaustion, depression, sleep disturbance, and self-rated ill health). They found that the mediators fully accounted for the relationships between leadership orientations and outcomes. 

Overall, research shows that support for the direct effect of leadership on employees’ stress and well-being is limited when mediators are included, that other leadership styles need to be considered besides the transformational one, and that different work characteristics may act as mediators.

### 1.2. Beneficial Advantages of Supervisor Development as a Preventive Intervention

Given the crucial role played by supervisors in the work stress process, their development has been proposed as an important kind of intervention available to organizations [1,5]. According to Kelloway and Barling [1], actions of this kind have different positive characteristics in preventing work stress:

- they can be classified as the most effective strategy of primary intervention (i.e., measures focused on reducing or eliminating the stressors);
- in spite of the tendency in organizations to consider occupational health psychology interventions as “nice to have” rather than as integral to the effectiveness of the organization [12], leadership development interventions are well accepted in industry and may mitigate some of the difficulties in implementing organization-level interventions;
- compared to other measures (e.g., job redesign, flexible work organization, etc.) leadership development is a cost-effective approach resulting in minimal disruption to the workplace;
- even if the intervention is intended to impact others (employees and their health), it may lead to positive results for the leaders as well, such as greater self-efficacy and well-being.

Moreover, given the active role of supervisors in implementing organizational interventions [13], leadership development could be a way to involve them in work stress prevention activities from the outset, thus facilitating the evaluation process.

In sum, leadership development can indeed be considered an organizational-level, cost-effective, and better-accepted intervention, with positive outcomes for both employees and supervisors. Research and applications in this area are increasing, but studies have rarely focused specifically on leader development with respect to work stress issues. 

A notable exception is the Health and Safety Executive framework mentioned above, which has been cited both for its theoretical [14] and practical [5] importance. 

### 1.3. Management Competencies for Preventing and Reducing Stress at Work

This approach was developed through a research project commissioned by the UK Health and safety Executive (HSE). Yarker et al. [3,4] provided three kinds of outcomes: a theoretical framework of management competencies and a link with the psychosocial work environment, a questionnaire aiming to measure the competencies, and a learning and development intervention for supervisors aiming to improve their competencies.

First, in reviewing the literature on leaders and employees’ well-being, Yarker et al. [3] noted that none of the various leadership models available focused on a comprehensive list of supervisors’ behaviours specific to the management of work-related stress. They therefore chose to focus on a competency framework (i.e., a set of behaviours) because it entails a language and format more accessible to organizations, better specifies expectations upon supervisors about the work stress issue, and facilitates the development of supervisors in terms of skills and behaviours. After extensive qualitative and quantitative work [4] they identified four important management competencies for the prevention of work-related stress. The first is being “respectful and responsible” (RR), which concerns the behaviours of a supervisor who shows integrity and is able to manage emotions. The second competency centres on “managing and communicating existing and future work” (MCW) and includes proactive work management, problem solving, and a participative approach. The third competency concerns “reasoning and managing difficult situations” (RDS), and includes the management of conflict, the use of organizational resources, and taking responsibility for resolving issues. The last competency is called “managing the individual within the team” (MIT) and includes being personally accessible and sociable, and showing an empathetic engagement.

It should be noted that the management competencies framework was strictly linked to the UK Health and Safety Executive Management Standards approach [15], a psychosocial risk assessment and prevention model which proposed supervisors’ development as a main preventive measure. In particular, the emerging framework was mapped onto the management standards definitions, evaluating the expected role of each competency for the psychosocial work environment. The authors concluded that the competencies were related to all the management standards (with the sole exception of “change”) and all the competencies mapped onto more than one management standard area. Thus, organizations could evaluate both psychosocial working conditions and the supervisors’ management competencies potentially impacting on those conditions by using an integrated framework and a standard set of measures.

Second, to measure the four competencies, a 66-item questionnaire (the SMCIT) was developed. The SMCIT can be used in the form of a self-evaluation exercise aimed at assessing and improving the supervisor’s own stress management competencies, or it can also be administered in a parallel form to the supervised employees, with the results used for upward feedback.

Third, a more complex learning and development intervention was designed to develop supervisors’ management competencies on the basis of the SMCIT results and upward feedback activity. It consists of a structured workshop directed at supervisors, aiming to explore the importance of supervisor behaviours, increase awareness of their own behaviours, and help them to foster positive behaviours.

However, despite these three important contributions, Toderi et al. [16] noted that no empirical research has been published on them, and both the framework and the questionnaire remain unevaluated. They suggested that the main problem was the high number of items in the SMCIT (66), which, given the low number of participants usually involved in these kinds of interventions, limits research and evaluation possibilities (e.g., exploring psychometric properties, adding measures of theoretically linked variables, etc.). Consequently, Toderi at al. developed a shorter 36-item version of the SMCIT in Italian, conducting a stringent psychometric evaluation of the tool [17]. The results showed that the postulated four-factor solution fitted employees’ data well, sustaining the validity and reliability of the short SMCIT. Second, they found that the four competencies show the expected relationships with measures of psychosocial work environmental factors, providing a first empirical confirmation of the theoretical framework.

### 1.4. Aims of the Study

Our purpose in the research reported in this paper was to advance this line of inquiry in two ways: (1) by providing further support for the 36-item version of the SMCIT; and (2) by exploring if and how supervisors’ stress-preventive competencies can affect employees’ affective well-being. 

First, given the high latent intercorrelations among the four management competencies (>0.80; see [17]), we postulated that such competencies could be modelled as indicators of a stress management competency meta-factor, which we used as the key construct for the subsequent analyses. Thus, as a preliminary step, we conducted a multilevel confirmatory factor analysis (CFA) using the four competencies scale scores to test whether a one-factor solution can fit the employees’ data. In this analysis, work group membership was used as a clustering variable. By doing this we provide for the first time a multilevel analysis of SMCIT data, which is a more correct approach than those previously adopted, since such data are naturally clustered in most studies and applications. Additionally, because self-assessments and assessments of others involve different processes and factors [18], we tested whether the same one-factor solution of the SMCIT would hold for the self-assessments provided by supervisors. Our first hypothesis was as follows:

**Hypothesis** **1.** The SMCIT data provided by employees (1a) and their supervisors (1b) would show the same one-factor solution, thus suggesting that the underlying model is configurationally equivalent.

Second, our intention was to provide further evidence that the line manager may affect employee stress and affective well-being, and that this happens mainly through an indirect process in which intervening factors are aspects of the psychosocial work environment experienced by employees (see, e.g., [2]). In other words, we postulated that the line manager, by enacting stress-preventive behaviours (i.e., those investigated by the SMCIT) contributes to creating a healthy psychosocial work environment (e.g., a manageable workload, a supportive social climate, good relationships), which in its turn relates to a higher level of affective well-being in employees. In this manner, we also tested the potential of an intervention model (i.e., developing management competencies), specifically conceived for primary prevention of work-related stress. An original feature of our research is that we tested the proposed chain of relationships by using multisource data: the stress prevention management competencies were estimated by using supervisors’ self-assessments, while employee data were used to operationalize the psychosocial work environment experienced by employees, and their affective well-being. The second hypothesis was the following:

**Hypothesis** **2.** The psychosocial work environment perceived by employees mediates the relationship between the supervisors’ stress-preventive management competency and employees’ affective well-being.

**Figure 1:**
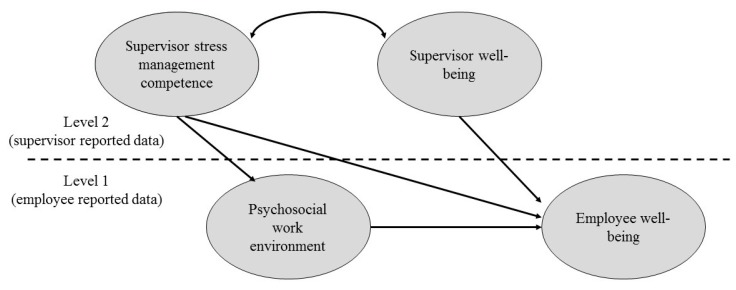
The study’s conceptual model.

Since supervisors’ affective well-being may be a particularly important variable in the context of the tested model, this was also included. Supervisor affective well-being may be related to the capacity of the supervisors to implement their stress-preventive management competencies. It could also influence the affective well-being of the supervised employees, for example directly through a cross-over contagion process [2,19]. Thus we believed it important to include supervisors’ affective well-being and to evaluate its role in the context of the tested model (Figure 1).

## 2. Materials and Methods 

### 2.1. Participants 

Various Italian organizations operating in different sectors were contacted and asked to participate in research on the role of supervisors’ management competencies for the improvement of employees’ well-being. In total, 16 organizations—mainly small to medium-sized—agreed to participate, involving overall 84 work teams and their supervisors. Employees and supervisors filled in a self-administered research questionnaire anonymously and on a voluntary basis. 

In total, 589 employees correctly filled in and returned the research questionnaire, with an average number of employees per group of 7.01. For 11 of the 84 groups the actual number of employees was not available to the researchers. For the remaining 73 groups, 527 employees out of a total of 1017 returned the questionnaire, giving an overall response rate of 51.8%. At an organizational level, the response rate varied between 42.7% and 55.5%.

The 589 employees were mainly men (52.1%), with 13.4% of the employees aged under 29; 28.9% between 30 and 39, 41.4% between 40 and 49, and 16.4% aged 50 or over. The mean working experience was 17.95 years (SD = 10.02; range 1–55) and the mean tenure in the team was 10.31 years (SD = 8.05; range 1–42).

The 84 supervisors were mainly male (73.9%). None of them were aged under 29 years, 13.4% were aged between 30–39, 47.8% between 40–49 and 38.8% were older than 50. Their mean working experience was 23.67 years (SD = 8.09; range 7–39) and the mean tenure in the team was 11.67 years (SD = 9.26; range 1–38). 

### 2.2. Procedure

The study was conducted in a slightly different fashion according to the organization involved. In all cases, supervisors were informed about the objective of the activity, and were assured that the results would be confidential. In some cases (depending on the organization’s availability), supervisors were also informed about the possibility, upon request, of sharing the main results with the organization (i.e., an entrepreneur or top management), and to discuss any potential needs for support or training activities. In one organization, the results were delivered to groups of supervisors in specific meetings.

Employees were informed about the activity mainly in written form (i.e., an email, a letter attached to the questionnaire). After the explanation of the aim of the activity (i.e., investigating the supervisors’ roles in increasing employees’ well-being at work) they were assured about the anonymity of their responses and that the results would be aggregated at a unit level, avoiding personal information (i.e., gender, age, etc.).

Supervisors and employees were also assured that single questionnaire data would be visible only to the external researchers, with only aggregated results and reports being available to organizational members. They were also provided with an email contact for any further information needed about the research.

Depending on the context, the questionnaire was administered online (i.e., through organizational intranet) or via a paper and pencil format. In the former case, employees were paired to their unit (i.e., supervisor) through a unit code that they were requested to insert at the beginning of the questionnaire. In the latter case, employees filled in the questionnaire during working hours and then put it in a box available in their working environment.

### 2.3. Measures 

*Management Competencies*. Use was made of the short 36-item version of the SMCIT [16,17], measuring each of the four competencies with nine items. Similarly to the original 66-item version, the questionnaire has two different forms: one intended for the self-assessment of supervisors (all items are prefixed by “I…”), and one for employees (“My supervisor….”). The supervisor/employee is requested to indicate her or his agreement with each of the presented statements on a five-point Likert scale (1—strongly disagree; 5—strongly agree). The employees’ version of the questionnaire is provided in Table 1. The psychometric properties of the tool, including its four-factor structure, have been verified in previous studies [16,17]. Cronbach’s alpha for the SMCIT and the other scales used are reported in Table 2. 

*Work environmental psychosocial factors*. These factors were investigated by using the Stress Management Indicator Tool (SMIT, [15,20]), which measures the following seven factors: demands, control, managerial and peer support, relationships, role, and change. A 25-item version of the SMIT developed by Edwards and Webster [21] and validated in Italian by Balducci et al. [22] was used. The validity of this shortened SMIT has been established (e.g., [22,23]). There is also evidence of invariance between the UK and Italian versions of the SMIT (see [24]). Items are rated on a 5-point Likert scale, varying from 1 (never or strongly disagree, according to specific items) to 5 (always or strongly agree). Example items are “I have unachievable deadlines” (demands, four items), “I have some say over the way I work” (control, four items), “I am given supportive feedback on the work I do” (managerial support, five items), “If work gets difficult, my colleagues will help me” (peer support, four items), “I am subject to bullying at work” (relationships, two items), “I am clear about the goals and objectives for my department” (role, three items) and “Staff are always consulted about change at work” (change, three items). Demands and relationships were reverse-scored before statistical analyses, so that higher scores on all the psychosocial factors indicated a better psychosocial environment.

*Job-related affective well-being*. This factor was measured using an Italian translation of the 12-item scale developed by Warr [25]. The scale consists of a list of 12 feelings, six positive (contented, calm, relaxed, enthusiastic, cheerful, optimistic) and six negative (depressed, tense, uneasy, gloomy, worried, miserable). Respondents (i.e., employees and supervisors) were asked to evaluate each feeling indicating how often, over the last month, their job had made them feel in that way (1—never to 5—always). The scale has been widely used [26] also in the Italian context [27,28]. Two six-item subscales were derived for the analyses, one measuring positive affective experiences and the other measuring negative affective experiences. 

### 2.4. Analysis

Hypothesis 1, that a one-factor solution would fit scale level data of the SMCIT, was tested by twice conducting confirmatory factor analysis (CFA), once for the employee data and again for supervisor data. In the case of employees, the analysis was a multilevel CFA, since group membership was taken into account and used as a clustering variable. Hypothesis 2, that the psychosocial work environment would mediate the relationship between an overall stress management competency and employees’ well-being, was tested by conducting multilevel structural equation modelling (MSEM), fitting a so-called 2-1-1 model [29]. Such terms describe a model where a level-2 main independent variable (in our case, the supervisor’s stress management competency) predicts a level-1 mediator (the psychosocial work environment), which in its turn predicts a level-1 dependent variable (employees’ well-being). Two versions of the same model shown in Figure 1 were tested, the difference between the two being the operationalization of the psychosocial work environment construct (see below). The supervisor stress management competence was measured by the four specific competencies investigated by the SMCIT, as reported by supervisors. By using the supervisors’ ratings on the SMCIT we avoided the pitfall of having all the data coming from the same source (i.e., the employees) and the associated problem of common method variance. The psychosocial work environment was defined in terms of a job content factor in one case, with demands and control as observed indicators, and in terms of a work context factor in a second case, with management and peer support, role, relationships, and change as manifest indicators. This differentiation of psychosocial work environmental factors is well established in the literature [30] and allows us to summaries the seven factors measured by the Stress Management Indicator Tool in two overall dimensions. Employees’ affective well-being was measured through the two indicators of positive and negative affective experiences. The same measures, as derived from the supervisors’ data, were used as indicators of supervisors’ affective well-being.

We adopted MSEM to test the main study hypothesis (i.e., Hypothesis 2), since MSEM has been shown to be superior to standard multilevel modelling (MLM) techniques when applied to mediation analysis [29]. Standard MLM techniques tend to conflate between- and within-level effects of level-l 1 variables with other level-1 variables [31]. On the contrary, MSEM separates the components within and between all variables, which allows for examination of direct and indirect effects at each level, including contextual effects across levels. It has been shown that MSEM dramatically reduces bias in the estimation of mediation effects in clustered data [29]. 

The main analyses were implemented using Mplus 7.4 (Muthén & Muthén, Los Angeles, CA, USA). Preliminary descriptive and correlational analyses were conducted using SPSS 22 (IBM, Armonk, NY, USA). 

## 3. Results

Descriptive statistics (Table 2) showed that the internal consistencies of all the used scales were at least adequate. The correlations between the different stress management competencies as self-assessed by the supervisors were positive and strong, varying from 0.49 to 0.81. There were also significant and moderate correlations between the stress management competencies self-assessed by the supervisors and their affective well-being, indicating that supervisors who reported stronger stress management competencies more often experienced positive affected states and less often experienced negative ones. At the employee level, there were positive and significant correlations between the stress management competencies attributed to the supervisors and factors of the psychosocial work environment, indicating that good stress management competencies go hand in hand with a better quality of the psychosocial work environment. Additionally, employees who attributed stronger stress management competencies to their supervisor also tended to report more often positive, and less often negative, affective experiences. In further analyses (not reported in Table 1) we found that there was a significant and positive relationship between each of the stress management competencies as self-assessed by the supervisor and the same competency attributed to them, on average, by their employees: respectful and responsible, *r* = 0.27, *p* < 0.05; managing and communicating work, *r* = 0.22, *p* = 0.05; managing difficult situations, *r* = 0.25, *p* < 0.05; managing the individual within the team, *r* = 0.30, *p* < 0.01. 

To test Hypothesis 1a, that the four competencies investigated by the SMCIT could be modelled as manifest indicators of a more general stress management competence (which we intended to use in further analyses), we conducted a multilevel CFA on the employees’ data (*n* = 589, distributed in 84 groups) where group membership was used as cluster variable. This analysis was a scale-level CFA. The intraclass correlation coefficient (ICC) for the four manifest indicators was high (i.e., >0.20 —see Preacher et al., 2011), varying from 0.23 (managing difficult situations) to 0.31 (managing the individual within the group), suggesting that there was considerable variability between the groups. Results of the analysis showed that the model fit the data very well: χ^2^(4) = 4.097, *p* = 0.39, CFI = 1.00; TLI = 1.00; RMSEA = 0.01; RMSR_within_ = 0.01, and RMSR_between_ = 0.02, with an average factor loading of 0.84 within levels (range: 0.79–0.88) and of 0.88 between levels (range: 0.75–0.96). This provided support for Hypothesis 1a. (χ^2^ = goodness-of-fit chi square; CFI = Comparative Fit Index; TLI = Tucker-Lewis Index; RMSEA = root mean square error of approximation; RMSRwithin = root mean square residual, within group (level 1); RMSRbetween = root mean square residual, between group (level 2)).

To test Hypothesis 1b, proposing that the same one-factor model of the SMCIT that emerged for employees would also hold for the supervisors’ data, we then ran a second CFA (*n* = 84). The results were as follows: χ^2^(2) = 7.513, *p* = 0.02; CFI = 0.99; TLI = 0.97; RMSEA = 0.18; and RMSR = 0.03; with an average factor loading of 0.82 (range: 0.62–0.93). There was less of a fit in this case, particularly as indicated by the RMSEA. However, it should be noted that the RMSEA is not reliable at low *n* and degree of freedom (df), with Kenny et al. [32] arguing that the RMSEA should not even be computed for low-df models. All this considered, we judged the results obtained as evidence in support of Hypothesis 1b.

To test Hypothesis 2, that the supervisor’s stress management competency would be related to the employees’ well-being through a better psychosocial work environment, we conducted multilevel structural equation modelling (MSEM), fitting two different versions of the model shown in Figure 1, as explained above. The ICC for all nine level-1 variables (i.e., employees’ negative and positive affective experiences and their report of the seven psychosocial work environmental factors: demand, control, supervisor and peer support, role, relationships, and change) was medium to high, ranging from 0.11 to 0.36 (average ICC: 0.23). The first model tested (see Figure 1—with supervisor and peer support, role, relationships, and change acting as indicators of the psychosocial work environment), obtained an adequate fit to the data: χ^2^(73) = 177.128, *p* < 0.001; CFI = 0.94; TLI = 0.92; RMSEA = 0.05; RMSR_Within_ = 0.052; and RMSR_Between_ = 0.071. All the observed variables loaded strongly on the hypothesized latent construct. For level 1 variables, this happened at both level 1 (individual level) and level 2 (group level). With regards the structural part of the model, at level 1 the psychosocial work environment reported by employees was significantly and positively related to their affective well-being (estimate: 0.705, *t* = 10.019, *p* < 0.001).

The parallel estimate at level 2 was also statistically significant (estimate: 1.012, *t* = 4.845, *p* < 0.001). This suggested that the hypothesized mediator (the psychosocial work environment experienced by employees) was related to the hypothesized dependent variable (employees affective well-being). Additionally, at level 2, the stress-preventive management competence of supervisors was strongly and positively related to the psychosocial work environment experienced by employees (estimate: 0.712, *t* = 4.772, *p* < 0.001), indicating that the hypothesized independent variable (supervisors stress-preventive management competence) was related to the hypothesized mediator (the psychosocial work environment reported by employees). However, the supervisors´ stress-preventive management competence was not directly related to the employees’ affective well-being (estimate: −0.171, t = −0.540, not significant. 

The supervisors’ affective well-being was significantly related to their stress-preventive management competence (estimate: 0.069, *t* = 2.377, *p* < 0.05); however, it was not significantly related to the employees’ affective well-being (estimate: 0.211, *t* = 0.777, ns). A test of the mediating role of the psychosocial work environment experienced by employees on the relationship between the supervisors’ stress-preventive management competence and employees’ affective well-being revealed significance (estimate: 0.720, *t* = 3.448, *p* < 0.01, 95% CI = 0.311–1.129). This supported Hypothesis 2, suggesting that the supervisors stress management competence influenced positively the employees’ affective well-being via the employees experience of a better psychosocial work environment (e.g., social support, relationships, etc.). 

The second model tested (with demand and control acting as indicators of the psychosocial work environment latent variable) also obtained an adequate fit to the data: χ^2^(31) = 58.752, *p* < 0.01; CFI = 0.97; TLI = 0.94; RMSEA = 0.039; RMSR_Within_ = 0.023; and RMSR_Between_ = 0.084. All the observed variables loaded strongly on the hypothesized latent construct. Structurally, at level 1 the psychosocial work environment reported by employees was significantly and positively related to their affective well-being (estimate: 1.303, *t* = 5.183, *p* < 0.001). The parallel estimate at level 2 was also statistically significant (estimate: 0.616, *t* = 2.554, *p* < 0.05). However, the only other significant relationship at level 2 was that between the stress-preventive management competence of supervisors and their affective well-being (estimate: 0.066, *t* = 2.292, *p* < 0.05). Crucially, the stress-preventive management competence of supervisors was not related to the psychosocial work environment experienced by employees (estimate: 0.342, *t* = 1.321, ns). Thus, the hypothesized mediation chain did not hold, which was not in line with Hypothesis 2. Overall, we found partial support for Hypothesis 2, with the mediating role of the psychosocial work environment being significant only when it was operationalized in terms of work contextual factors (see Table 3 for a summary of the results obtained). 

Finally, we again ran the two MSEM analyses including employees’ gender and age, with the latter categorized as 40–49 years versus others. These variables were modelled as factors influencing employees’ affective well-being. However, the main results did not change.

## 4. Discussion

Although the HSE framework on stress-preventive management competencies and the SMCIT questionnaire have been described as examples of good practice in this area [5], to the best of our knowledge no research has empirically investigated the effectiveness of the framework after its development. Because the action inspired by the framework has supervisors’ competencies development activities as an outcome, it is crucial to understand if and how the targeted managerial competencies are related to employees’ wellbeing. The present study aimed to provide initial evidence on this matter.

Our first hypothesis focused on the SMCIT questionnaire and provided further support for its validity, studying employees’ data at a group level (Hypothesis 1a) and, additionally, using supervisors’ scores (Hypothesis 1b). The 36-item SMCIT has been found to be a valid and reliable instrument using employees’ scores at an individual level [16,17]. However, SMCIT data are typically clustered, and this clustering structure is crucial for the following interventions, which are carried out at group level (i.e., to provide upward feedback to supervisors). Our multilevel analysis supported the strong intercorrelations between the four SMCIT competencies that previously emerged [17], and it showed that the four competencies may be thought of as being influenced by a meta-competence factor. This is compatible with the idea of a second-order factor solution, where individual items load on their respective first-order factors and first-order factors load on a second-order meta-factor. The obtained results are not inconsistent with previous results on the tool [17], rather they complement them, offering a valid alternative solution which permits to derive an overall score on the SMCIT. This alternative solution may be useful in future research aimed at expanding the nomologial network of the SMCIT, for example by investigating its relationship with additional outcome variables (sickness absence, turnover, etc.). Secondly, in line with Hypothesis 1b, we provided first support for the supervisors’ version of the questionnaire, showing that the same factor solution found for employees also held for the supervisors’ data. This evidence—which has never been provided before—is important, since interventions based on the SMCIT could involve a comparison between supervisors’ scores and employees’ scores, thus assuming that the two versions of the tool have the same underlying structure. However, this cannot be taken for granted, since self-ratings and ratings of others imply processes affected by different factors: for example, ratings of leadership by employees reflect not only the leader’s actual behaviour but also the rater’s cognitive schemas on effective leadership [18]. Thus, although the results reported in this study should be treated with caution since they were based on only 84 supervisors, they do provide encouraging evidence that the two versions of the SMCIT questionnaire have the same underlying structure. 

Our second hypothesis, the crucial hypothesis of the study, focused on the evaluation of the SMCIT management competencies in predicting employees’ affective well-being. The mediating role of the psychosocial work environment was confirmed for the work context factors, but not for the factors related to the content of the job (i.e., demands and control). The results provide partial support for the management competency framework, showing that supervisors’ behaviours (as measured by the 36-item SMCIT) relate to employees’ affective well-being by means of their relationship with contextual factors of work. Our results therefore replicate and extend previous research in this area.

We extended the type of supervisors’ behaviours that can be considered of interest for employees’ well-being through their relationship with the psychosocial work environment. The role of transformational leadership has been previously documented in this regard [9]. Lornudd et al. [10] extended the results to alternative leadership models (i.e., production-, employee- and change-oriented leadership) and Karanika-Murray et al. [33] found a mediating role of leader-member exchange (LMX) factors. These results support the idea that more than one leadership theory may be of interest for understanding employees’ affective well-being. Our study provides support for a comprehensive framework of supervisors’ management behaviours specifically tailored to work stress and well-being. It should be noted that the management competencies framework was mapped by Yarker et al. [4] onto other management or leadership frameworks relevant to employees’ well-being, including five transformational leadership frameworks and seven additional management models (see [4]). The result was that, even if all four stress management competencies investigated by the SMCIT were included in at least one of the leadership frameworks considered, no framework covered all four competencies.

Close attention should also be paid to the specific psychosocial factors studied when assessing the preventive role of leadership. In particular, Lornudd et al. [10] empirically supported the mediating role of demands and control in the relationship between leadership and employees’ well-being, which is at odds with the results of our study. This difference may be explained by the fact that Lornudd et al. used demands and control as separate factors in their regression analyses, which is a conceptualization different from the one used in the present study. In fact, while in Lornudd et al. the shared variance between demands and control was statistically removed from the analyses, in the present study such variance constituted the crucial mediating factor.

A further contribution of the present study in extending previous research in this area is linked to methodology. The leadership psychosocial work environment–well-being causal path was previously supported by Nielsen et al. [9], who found limited evidence for a direct effect of leadership behaviour on employee well-being. In the present study we have provided further support for the mediation model by adopting a multilevel approach and using multisource data (i.e., supervisors and employees). In particular, we note that the use of multisource data for some of the links of the hypothesized model is quite crucial for ruling out the role of common method variance. Thus, the importance of the indirect path in the relationship between leadership and employees’ affective well-being is strengthened by the present study. Additionally, we also controlled for supervisors’ affective well-being in our model, which is a further original feature of the present study. Supervisors’ affective well-being was not directly related to employees’ affective well-being, but it was positively related to the supervisors’ overall stress management competence. This suggests that more complex chains of relationships could be tested in future research with appropriate data: for instance, the indirect influence of supervisors’ affective well-being on employees’ affective well-being via supervisors’ stress management competence.

### Methodological Issues and Limitations

The most important limitation of the present study is the cross-sectional nature of the data available. It is well known that cross-sectional data may introduce significant biases into the estimation of mediation [34] and that three-wave data are beneficial. Thus, although the most original relationships in the tested model, particularly those involving stress-preventive management competence, were tested by using multisource data that also took their clustered organization into account, future research should implement longitudinal research. Only longitudinal research, ideally with a multisource and multilevel design and with carefully planned time lags between waves (see [35,36]), could provide robust evidence on the true nature of the relationships found in the present study. 

A methodological issue and a further potential limitation of the present study is that an overall management competence was modelled with the SMCIT data and used in the mediation model tested. A preliminary CFA revealed that the SMCIT data were compatible with this conceptualization. Previous research [17] has also shown the strong intercorrelations between the four SMCIT competencies. However, this conceptualization could have covered differential relationships between the four SMCIT competencies and the employees’ data. It could be, for example, that the adoption of a more fine-grained view on stress-preventive competencies would reveal the mediating role of job content psychosocial factors in the context of the hypothesized mediation model, which was not supported in the present study. Similarly, a more fine grained view on psychosocial factors could be adopted in future studies, for example by differentiating single factors (i.e., demands, control, support, etc.) and testing their mediating role in the relationship between supervisor management competency and employee well-being.

A further important limitation concerns the small sample of supervisors available for the CFA on supervisor data. Structural equation modelling techniques (including CFA) work best with large datasets (see [37]). However, the number of cases per parameter should also be considered, and in our case this was within the suggested range of 5/10 cases per parameter [37]. Additionally, recent simulation studies suggest that with simple models such as the one we tested, small samples (*n* = 70) may be enough to obtain reliable estimates [38,39]. Having said that, we believe that it is imperative to replicate the results we obtained in the present study with larger samples of supervisors.

## 5. Conclusions

The study provides further support to the validity of the 36-item SMCIT [16,17] and, for the first time, empirical evidence for the adequacy of the Management Competencies for Preventing and Reducing Stress at Work framework [3]. 

Because the questionnaire and the framework are strictly linked to the UK Health and Safety Executive Management Standards approach, organizations could evaluate both psychosocial working conditions and the supervisors’ management competencies, potentially impacting on those conditions by using a standard set of measures with a limited number of items (see [17]). Such data can be used to conduct a structured learning and development intervention divided into two phases: an upward feedback report, and a structured workshop organized for supervisors, so that they can explore the importance of supervisor behaviours for the employees’ psychosocial work environment, increase awareness of their own behaviours, and develop positive behaviours. 

## Figures and Tables

**Table 1:** The 36-item SMCIT (In parentheses the original item number in the 66-item version, Yarker et al. [4]).

Competency	Items
Respectful and responsible	1. Does not speak about team members behind their backs (50)
	2. Is consistent in his or her approach to managing (13)
	3. Creates unrealistic deadlines for delivery of work (4)
	4. Is honest (51)
	5. Acts calmly in pressured situations (45)
	6. Imposes “my way is the only way” (20)
	7. Treats me with respect (53)
	8. Passes on his or her stress to me (46)
	9. Shows a lack of consideration for my work-life balance (36)
Managing and communicating existing and future Work	10. When necessary, will stop additional work being passed on to me (2)
	11. Follows up problems on my behalf (5)
	12. Gives me the right level of job responsibility (18)
	13 Reviews processes to see if work can be improved (10)
	14. Is indecisive at decision-making (8)
	15. Encourages participation from the whole team (22)
	16. Prioritises future workloads (11)
	17. Deals with problems as soon as they arise (9)
	18. Correctly judges when to consult employees and when to make a decision (23)
Reasoning and managing difficult situations	19. Deals objectively with employee conflicts (37)
	20. Seeks help from occupational health when necessary (64)
	21. Supports employees through incidents of abuse (38)
	22. Deals with employee conflicts head on (39)
	23. Seeks advice from other managers when necessary (65)
	24. Follows up conflicts after resolution (40)
	25. Acts as a mediator in conflict situations (43)
	26. Uses HR as a resource to help deal with problems (66)
	27. Makes it clear he or she will take ultimate responsibility if things go wrong (59)
Managing the individual within the team	28. Is available to talk to when needed (29)
	29. Is willing to have a laugh at work (54)
	30. Takes an interest in my life outside work (61)
	31. Returns my calls/emails promptly (30)
	32. Socialises with the team (55)
	33. Tries to see things from my point of view (62)
	34. Prefers to speak to me personally rather than use email (31)
	35. Brings in treats (56)
	36. Makes an effort to find out what motivates me at work (63)

Note: SMCIT: Stress Management Competencies Indicator Tool; HR: Human Resources.

**Table 2:** Descriptive statistics and intercorrelations between the study variables.

Research Variables	Mean (SD) Level 1 ^1^	Mean (SD) Level 2 ^2^	1	2	3	4	5	6	7	8	9	10	11	12	13
1. Respectful/responsible	3.72 (0.76)	3.98 (4.63)	0.85/0.68	0.63 ***	0.50 ***	0.49 ***	−0.44 ***	0.25 *							
2. Managing and communicating work	3.54 (0.74)	4.02 (0.46)	0.70 ***	0.87/0.78	0.81 ***	0.76 ***	−0.37 **	0.28 *							
3. Managing difficult situations	3.46 (0.82)	3.99 (0.61)	0.64 ***	0.79 ***	0.91/0.88	0.77 ***	−0.31 **	0.33 **							
4. Managing individual within the team	3.61 (0.75)	3.96 (0.54)	0.69 ***	0.77 ***	0.75 ***	0.88/0.82	−0.28 *	0.27 *							
5. Negative affective experiences	2.38 (0.83)	2.33 (0.67)	−0.42 ***	−0.38 ***	−0.35 ***	−0.32 ***	0.86/0.79	−0.43 ***							
6. Positive affective experiences	3.22 (0.82)	3.48 (0.67)	0.48 ***	0.56 ***	0.56 ***	0.52 ***	−0.64 ***	0.87/0.80							
7. Demands	3.88 (0.74)		0.42 ***	0.28 ***	0.25 ***	0.28 ***	−0.46 ***	0.37 ***	0.77						
8. Control	3.43 (0.83)		0.37 ***	0.31 ***	0.26 ***	0.26 ***	−0.29 ***	0.38 ***	0.32 ***	0.84					
9. Supervisor support	3.70 (0.80)		0.68 ***	0.79 ***	0.73 ***	0.75 ***	−0.36 ***	0.54 ***	0.36 ***	0.36 ***	0.84				
10. Coworker support	3.89 (0.72)		0.38 ***	0.47 ***	0.46 ***	0.42 ***	−0.29 ***	0.41 ***	0.28 ***	0.31 ***	0.61 ***	0.84			
11. Relationship	4.50 (0.81)		0.48 ***	0.41 ***	0.38 ***	0.31 ***	−0.44 ***	0.40 ***	0.33 ***	0.35 ***	0.48 ***	0.38 ***	0.84		
12. Role	3.98 (0.83)		0.41 ***	0.47 ***	0.43 ***	0.41 ***	−0.26 ***	0.48 ***	0.33 ***	0.36 ***	0.50 ***	0.35 ***	0.29 ***	0.80	
13. Change	3.23 (0.86)		0.50 ***	0.63 ***	0.60 ***	0.59 ***	−0.37 ***	0.56 ***	0.37 ***	0.35 ***	0.73 ***	0.52 ***	0.36 ***	0.55 ***	0.76

Note. ^1^ Level 1 = employee level. ^2^ Level 2 = supervisor level. Supervisor data are reported above the diagonal, including self-assessment of stress management competencies (variables 1–4) and affective well-being (variables 5–6). Employee data are reported below the diagonal, including the assessment of the supervisor stress management competencies (variables 1–4), employee affective well-being (variables 5–6) and indicators of the psychosocial work environment (variables 7–13). Cronbach’s alpha is reported on the main diagonal—where two alpha values appear, the first refers to employee level data, and the second to supervisor level data. * *p* < 0.05, ** *p* < 0.01, *** *p* < 0.001.

**Table 3:** Summary of the results obtained in relation to the tested mediation models.

Coefficients	Model 1 ^a^	Model 2 ^b^
Model fit indeces		
Chi-square	177.128	58.752
Degrees of freedom	73	31
CFI	0.94	0.97
TLI	0.92	0.94
RMSEA	0.05	0.04
RMSR_within_	0.05	0.02
RMSR_between_	0.07	0.08
Path		
SSMC-SAWB	0.069 *	0.066 *
SAWB > EAWB	0.211 ns	0.438 ns
SSMC > PWE	0.712 ***	0.342 ns
SSMC > EAWB	−0.171 ns	−0.227 ns
PWE > EAWB (Level 1 estimate)	0.705 ***	1.303 ***
PWE > EAWB (Level 2 estimate)	1.012 ***	0.616 *

^a^ In Model 1, the psychosocial work environment was operationalized by using work context factors (supervisor and peer support, role, relationships, and change). ^b^ In Model 2, the psychosocial work environment was operationalized by using job content factors (demand and control). SSMC = supervisor stress management competence; SAWB = supervisor affective well-being; EAWB = employee affective well-being; PWE = psychosocial work environment. * *p* < 0.05, ** *p* < 0.01, *** *p* < 0.001. CFI: Comparative Fit Index; TLI: Tucker-Lewis Index.
